# A Recombinant Porcine Reproductive and Respiratory Syndrome Virus Stably Expressing a Gaussia Luciferase for Antiviral Drug Screening Assay and Luciferase-Based Neutralization Assay

**DOI:** 10.3389/fmicb.2022.907281

**Published:** 2022-05-13

**Authors:** Yanhua Li, Cicheng Ren, Chenxi Li, Yihong Xiao, Yanyang Zhou

**Affiliations:** ^1^College of Veterinary Medicine, Yangzhou University, Yangzhou, China; ^2^Comparative Medicine Research Institute, Yangzhou University, Yangzhou, China; ^3^Jiangsu Co-Innovation Center for Prevention and Control of Important Animal Infectious Diseases and Zoonosis, Yangzhou, China; ^4^College of Animal Science and Veterinary Medicine, Shandong Agricultural University, Tai’an, China

**Keywords:** porcine reproductive and respiratory syndrome virus, infectious cDNA clone, gaussia luciferase, antiviral drug screening, serum neutralization assay

## Abstract

The reverse genetics system is a valuable tool in the virological study of RNA viruses. With the availability of reverse genetics, the porcine reproductive and respiratory syndrome virus (PRRSV) has been utilized as a viral vector for the expression of foreign genes of interest. Here, we constructed a full-length cDNA clone of a highly pathogenic PRRSV (HP-PRRSV) TA-12 strain. Using this cDNA clone, we generated a reporter virus expressing a gaussia luciferase (Gluc) *via* an additional subgenomic RNA between ORF7 and 3′UTR. This reporter virus exhibited similar growth kinetics to the wild-type (WT) virus and remained genetically stable for at least ten passages in MARC-145 cells. In cells infected with this reporter virus, the correlation between the expression levels of Gluc in culture media and the virus titers suggested that Gluc is a good indicator of the reporter virus infection. With this reporter virus, we further established the Gluc readout-based assays for antiviral drug screening and serum neutralizing antibody detection that exhibited comparable performance to the classical assays. Taken together, we established a reverse genetics system of HP-PRRSV and generated a novel reporter virus that could serve as a valuable tool for antiviral drug screening and serum neutralizing antibody detection.

## Introduction

Porcine reproductive and respiratory syndrome (PRRS) which is one of the most economically important swine diseases mainly causes respiratory diseases to newborn and growing pigs, and reproductive failure in pregnant sows. The causative agent of PRRS is the porcine reproductive and respiratory syndrome virus (PRRSV) which belongs to the *Arteriviridae* family of the order *Nidovirales*. Previously, PRRSV was classified into two genotypes (type 1 and type 2) that have been promoted to two species (*PRRSV-1* and *PRRSV-2*).^1^ In China, the dominant strains are PRRSV-2, although PRRSV-1 strains have been sporadically isolated. PRRSV genome is a capped positive-sense RNA molecule that is about 15 kb in length, contains a 5′ untranslated region (5′UTR), 11 open reading frames (ORFs), and a 3′UTR followed by a poly(A) tail. The 5′ end 75% genome encodes at least 14 nonstructural proteins (nsp1α/β, nsp2N, nsp2TF, nsp2 ~ nsp6, nsp7α/β, and nsp8 ~ nsp12), while the rest 3′ end genome encodes structural proteins through a set of subgenomic RNAs that share the 5′ and 3′ ends (Fang and Snijder, 2010; Fang et al., 2012; Lunney et al., 2016). Since PRRSV RNA-dependent RNA polymerase (RdRp) lacks proofreading ability, the PRRSV genome has an extremely high mutation rate (Hanada et al., 2005; Murtaugh et al., 2010) which leads to the emergence of novel PRRSV strains almost every few years during the past 30 years, such as the HP-PRRSV strains in Asian countries (Tian et al., 2007), NADC30-like strains in China (Zhao et al., 2015; Zhou et al., 2015; Ji et al., 2016; Li et al., 2016), and NADC34-like strains in China (Xu et al., 2022). Although vaccination is the most effective strategy for PRRS control, commercial vaccines provide limited protection against heterologous infection (Bai et al., 2016; Zhou et al., 2017; Chai et al., 2020; Chen et al., 2021). Besides, the restricted biosafety measures and point of care diagnostics are critical for PRRS control and eradication in the future.

The first reverse genetics for PRRSV-1 was established in 1998 (Meulenberg et al., 1998). Since then, many infectious cDNA clones for both PRRSV species have been constructed and become a valuable tool in PRRSV investigations as summarized by Chaudhari and Vu (2020). With the availability of reverse genetics, PRRSV has been explored as a viral vector to express a foreign gene of interest, such as reporter genes (Fang et al., 2006; Guo et al., 2016; Huang et al., 2018) for tracking viral infection and antigens of other pathogens for vaccine development (Pei et al., 2009; Zheng et al., 2010; Gao et al., 2018). Two main strategies have been employed to deliver the foreign gene, including as a fusion protein within nsp2 and as an additional subgenomic RNA inserted at three potential locations in the PRRSV genome: between ORF1b and ORF2a, between ORF4 and ORF5a, and between ORF7 and 3′UTR (Pei et al., 2009; Wang et al., 2013, 2020; Tian et al., 2017). The intergenic junctions between ORF1b and ORF2a and between ORF7 and 3′UTR are the most commonly used locations for gene insertion. To date, several recombinant reporter viruses expressing fluorescent proteins (EGFP, RFP, and *Renilla* luciferase) generated through these strategies have been utilized for antiviral drug screening and serum neutralization assay (SNA; Sang et al., 2012; de Wilde et al., 2013; Wang et al., 2013). The classical SNA for PRRSV neutralizing antibody detection is the fluorescent focus unit reduction-based test that is time-consuming and laborious (Ostrowski et al., 2002). By contrast, the SNA using reporter viruses could be less time-intensive and applicable for high-throughput format.

Since Gaussia luciferase (Gluc) from the marine copepod *Gaussia princeps* is naturally secreted from mammalian cells in an active form (Tannous et al., 2005), it can be detected in culture media without cell lysis. In addition, Gluc is over 1,000-fold more sensitive than the commonly used firefly luciferase and *Renilla* luciferase. Due to its small size, unique thermal stability, and genetically encoded secretion system, Gluc has been used as a reporter to monitor viral infection (Nishiyama et al., 2019; Wang et al., 2019; Zhang et al., 2021; Lucke et al., 2022). In this study, we constructed an infectious cDNA clone of the HP-PRRSV TA-12 strain using the DNA-based transfection approach. With this reverse genetics, a recombinant virus expressing Gluc (rTA-Gluc2) was constructed and rescued. This reporter virus exhibited similar growth kinetics as the WT virus and remained genetically stable for at least ten passages *in vitro*. Of note, the expression levels of Gluc in culture media of the reporter virus-infected cells were correlated with virus titers. Using this reporter virus, we established Gluc readout-based antiviral drug screening assay and SNA for HP-PRRSV.

## Materials and Methods

### Cells, Viruses, Antibodies, and Compounds

MARC-145 cells (ATCC) were cultured in Modified Eagle Medium (MEM; Sigma-Aldrich, St. Louis, MO, United States) containing 10% fetal bovine serum (FBS; Sigma-Aldrich, St. Louis, MO, United States) and 1% penicillin–streptomycin (Thermo Fisher Scientific, Waltham, MA, United States). BHK-21 cells purchased from ATCC were maintained in Dulbecco’s Modified Eagle Medium supplemented with 10% FBS (Gemini Bio, West Sacramento, CA, United States) and 1% penicillin–streptomycin (Thermo Fisher Scientific, Waltham, MA, United States). The HP-PRRSV TA-12 strain (GenBank Accession No. HQ416720.1) was purified by plaque assay in MARC-145 cells, and the complete genome of one plaque was determined by the Sanger sequencing method and deposited to GenBank under accession No. MZ399801. A monoclonal antibody (clone 4A5) against PRRSV N protein was purchased from MEDIAN Diagnostics, Korea. IFN-α2b (Sangon Biotech, Shanghai, China), ribavirin (Sigma-Aldrich, St. Louis, MO, United States), 5-Fluorouracil (MedChemExpress, Shanghai, China), and Chloroquine (MedChemExpress, Shanghai, China) were used in this study. Coelenterazine h (Maokang Biotechnology, Shanghai, China) was dissolved in acidified methanol to a concentration of 5 mg/ml, and aliquots were stored at −80°C.

### Assembly of a Full-Length cDNA Clone of HP-PRRSV TA-12 Strain by Homologous Recombination *in vitro*

**Figure 1:**
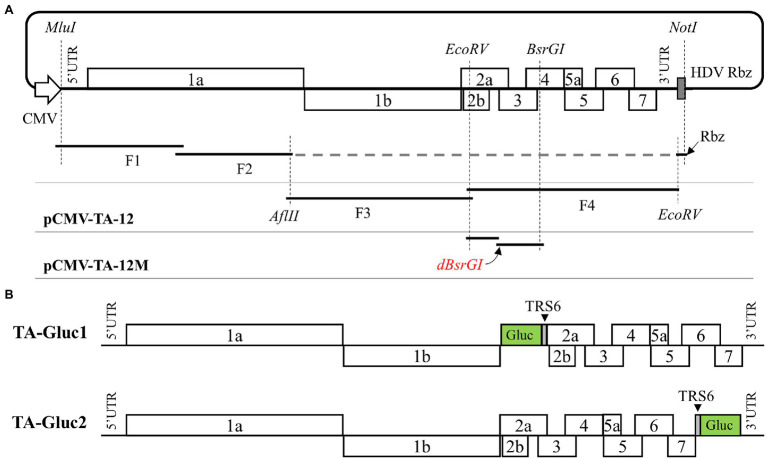
Construction of an infectious cDNA clone of HP-PRRSV TA-12 strain and the reporter viruses expressing a Gluc. **(A)** An infectious cDNA clone of TA-12 assembled through homologous recombination *in vitro* as described in the “Materials and Methods.” **(B)** Two cDNA clones were designed to express a Gluc *via* two strategies, respectively. In TA-Gluc1 construct the Gluc coding sequence followed by a PRRSV TRS6 was inserted into pCMV-TA-12 M between ORF1b and ORF2a, while in TA-Gluc2 construct a PRRSV TRS6 followed by the Gluc coding sequence was inserted between ORF7 and 3′UTR.

Initially, a synthesized DNA fragment containing a human cytomegalovirus immediate-early promoter (CMV), the unique restriction enzyme sites (*MluI, AflII*, and *EcoRV*), partial 3′UTR of TA-12, and a hepatitis D virus ribozyme (HDV Rbz) were cloned into pACYC177 plasmid to create the pCMV-TA-12-vector. The complete genomic cDNA of TA-12 was divided into four overlapping fragments and inserted into the pCMV-TA-12-vector through a two-step cloning strategy based on homologous recombination technology. Briefly, viral RNA of TA-12 extracted with the FastPure Viral DNA/RNA Mini Kit (Vazyme Biotech, Nanjing, China) was used as a template to synthesize cDNA using the Superscript IV first-strand synthesis system (ThermoFisher Scientific, Waltham, MA, United States), and four fragments (F1 ~ F4) covering the complete genome of TA-12 were amplified using the Q5 high-fidelity DNA polymerase (NEB, Ipswich, MA, United States) and primers listed in Table 1. To facilitate homologous recombination, the neighboring individual DNA fragments share around 20 nucleotide acids. As illustrated in Figure 1A, F1 and F2 were inserted into the pCMV-TA-12-vector which was linearized with restriction enzymes (*MluI* and *AflII*) using the NEBuilder HiFi DNA Assembly Master Mix (NEB, Ipswich, MA, United States) in the first round homologous recombination. Similarly, F3 and F4 were assembled into a full-length cDNA clone named pCMV-TA-12. To distinguish the rescued virus from the wild-type (WT) virus, a genetic marker was introduced by the disruption of the *BsrGI* restriction site in ORF4 using primers in Table 1. Finally, the infectious cDNA clone of TA-12 was designated as pCMV-TA-12 M.

**Table 1:** The primers used in this study.

**Name**	**Sequence**	**Usage**
F1-F	AGGTCTATATAAGCAGAGCTACGCGTTAATAGCATGACGTATAGGTGTTGGC	Construction of the full-length cDNA clone
F1-R	TGCGTAGCAGGATCACTAAG	
F2-F	GCTTGTGATGCGTCCAAG	
F2-R	TAATTGAATAGGAATTCGATATCCTTAAGTTCATTACCACCTGTAACG	
F3-F	CGTTACAGGTGGTAATGAACTTAA	
F3-R	ATGGCCAAAAATATGATGATATCAAC	
F4-F	TGGTGTCCATTGTTGATATCAT	
F4-R	ACTTTACCCCCACACGGT	
TA-12-EcoRV-F	GAATTTCTGGTGTCCATTGTTG	Introduction of a genetic marker into cDNA clone
TA-12-dBsrGI-R	GGAGGAATGGACAGCTATTAGCC	
TA-12-dBsrGI-F	GGCTAATAGCTGTCCATTCCTCCATA	
TA-12-BsrGI-R	ACATTGGCAGTGATGGTG	
TA-12-BsrGI-F	CACGGCGATAGGGACGCC	Construction of pCMV-TA-Gluc2
TA-12-N/TRS6-R	AACTCTGGTTAAAGGGGTTGCCGCGGAACTCATGCTGAGGGTGATGCTGT	
TA-12-TRS6/Gluc-F	CAACCCCTTTAACCAGAGTTTCAGCGGAACAATGGGAGTCAAAGTTCTGTTTGCC	
TA-12-Gluc/UTR-R	TGCCAGCCCATTAGTCACCACCGGCCCC	
TA-12-Gluc/UTR-F	TGGTGACTAATGGGCTGGCATTCTTTGGC	
TA-12-NotI-R	CCGGAAGGGCTTAAAGCGG	
TA-12-BsaBI-F	CAAACACACCTGGGGATTT	Construction of pCMV-TA-Gluc1
TA-12-nsp12/Gluc-R	TGACTCCCATTTCAATTCAGGCCTAAAGTTGG	
TA-12-nsp12/Gluc-F	CTGAATTGAAATGGGAGTCAAAGTTCTGTTTG	
TA-12-Gluc/TRS6-R	AACTCTGGTTAAAGGGGTTGCCGCGGAACTTAATTAATTAGTCACCACCGGCCCC	
TA-12-TRS6/GP2-F	CAACCCCTTTAACCAGAGTTTCAGCGGAACAATGAAATGGGGTCTATGCAAAGCC	
PRRSV-F1	GTCAATCAGCTGTGCCA	RT-PCR and DNA sequencing
PRRSV-R	CCCTAATTGAATAGGTGRCTT	

### Construction of cDNA Clones Containing an Expression Cassette of Gluc Gene

The infectious cDNA of TA-12 was developed as a viral vector to express Gluc through an additional subgenomic RNA. The expression cassette of Gluc was inserted into the cDNA clone at two sites, ORF1b/ORF2a and ORF7/3’UTR, respectively. As illustrated in Figure 1B, in the TA-Gluc1 clone, the Gluc coding sequence fused with a TRS6 sequence (gttccgcggcaacccctttaaccagagtttcagcggaaca) at the 3′ end was inserted between ORF1b and ORF2; in the TA-Gluc2 clone, the Gluc coding sequence with a TRS6 sequence at 5′ end was inserted between ORF7/3′UTR. Those two cDNA clones were assembled through homologous recombination using the NEBuilder HiFi DNA Assembly Master Mix (NEB, Ipswich, MA, United States) per the manufacturer’s instructions.

### Recovery of Recombinant Viruses

BHK-21 cells at ~80% confluence in a 12-well culture plate were transfected with 2 μg of a full-length cDNA clone using Lipofectamine^®^ 3,000 transfection reagent (ThermoFisher Scientific, Waltham, MA, United States) according to the manufacturer’s instructions. At 2 days post-transfection (dpt), culture supernatant was harvested to infect MARC-145 cells seeded in a 12-well culture plate 2 days ahead. At the same time, cell monolayers were fixed with ice-cold methanol for 20 min at −20°C and then stained with a mAb against N protein. Cells were monitored daily for the appearance of cytopathic effect (CPE) under the IX73 epifluorescence microscope (Olympus). Around 4 days post-infection (dpi), the culture supernatant was harvested as P1 virus and stored at −80°C, and then further passaged in MARC-145 cells at least ten times.

### Viral Growth Curve

The confluent MARC-145 cells in a 24-well culture plate were inoculated with rTA-12 M or rTA-Gluc at a multiplicity of infection (MOI) of 0.01. Cells were incubated with virus supernatant diluted with MEM for 1 h, then washed twice with 1×PBS, and cultured with 1 ml MEM supplemented with 2% FBS (Sigma-Aldrich, St. Louis, MO, United States). Virus supernatants were harvested at 0, 12, 24, 36, 48, 60, and 72 h post-infection (hpi) for virus titration by TCID_50_, and Gluc levels in virus supernatants were evaluated by luciferase assay. The viral growth curves were created with GraphPad Prism 8.

### Plaque Assay

The confluent monolayers of MARC-145 cells in 12-well culture plates were inoculated with 10-fold diluted rTA-12 M or rTA-Gluc2, respectively. Cells were incubated with virus supernatant diluted with MEM for 2 h, then washed twice with 1×PBS, and then overlaid with 5 ml of MEM containing 2% FBS (Sigma-Aldrich, St. Louis, MO, United States) and 1% UltraPure^™^ Low Melting Point Agarose (ThermoFisher Scientific, Waltham, MA, United States). At 3 dpi, the cells were fixed with 4% paraformaldehyde and stained with 0.1% crystal violet to visualize plaques.

### The Genetic Stability of rTA-Gluc *in vitro*

The reporter virus, vTA-Gluc2, was serially passaged ten times in MARC-145 cells. The genetic stability of rTA-Gluc2 was evaluated by checking the coding sequence of Gluc and the expression of Gluc. To check viral genomic sequence, viral RNAs of P5 and P10 were extracted using the FastPure Viral DNA/RNA Mini Kit (Vazyme Biotech, Nanjing, China). The viral genomic region of vTA-Gluc2 covering was amplified with primers listed in Table 1 using HiScript II One-Step RT-PCR Kit (Vazyme Biotech, Nanjing, China). The PCR product was purified and sent for DNA sequencing by GENEWIZ. We also compared the expression dynamic of Gluc through viral growth curves. MARC-145 cells were infected with P3, P5, and P10 viruses at an MOI of 0.01. Virus supernatants harvested at 0, 12, 24, 36, 48, 60, and 72 hpi were subject to luciferase assay.

### Indirect Immunofluorescence Assay

Cell monolayers were fixed with ice-cold methanol and then air-dry. For the detection of N protein expression, cells were incubated with a mAb (4A5) at 37°C for 1 h. After five washes, the cell monolayers were stained with Alexa 488-conjugated goat anti-mouse IgG(H + L; Jackson ImmunoResearch Inc., West Grove, PA, United States). Cell nuclei were counterstained with 4′,6-diamidino-2-phenylindole (DAPI) solution (Solarbio Life Sciences, Beijing, China) for 5 min at room temperature. After extensive washes with 1×PBS, fluorescent images were captured with the IX73 epifluorescence microscope (Olympus).

### Luciferase Assay

The gaussia luciferase assay was performed as described previously (Tannous, 2009). Coelenterazine h was diluted to 20 μM with PBS supplemented with 5 mM NaCl, pH 7.2, and incubated at room temperature in the dark for 30 min. To measure the gaussia activity, mix 50 μl of 20 μM coelenterazine h with 20 μl sample in a white plate and acquire photon counts for 10 s using a plate reader.

### *In vitro* Cytotoxicity Assay

The cytotoxicity to MARC-145 cells was evaluated for ribavirin, 5-Fluorouracil, and Chloroquine using the CCK-8 Cell Counting Kit (Vazyme Biotech, Nanjing, China) according to the manufacturer’s instructions. Briefly, MARC-145 cells were seeded in 96-well tissue culture plates and incubated at 37°C for 2 days. 100 μl compounds per well at the indicated concentrations were added to MARC-145, and four replicates for each concentration. At 24 h post-treatment, the cell monolayers were washed with 1×PBS and incubated with 100 μl of one to ten diluted colorimetric reagent for 1 h. Measurements from compound-treated or vehicle-treated cells were normalized against those from untreated cells. The half-maximum cytotoxic concentration (CC50) was calculated using Graphpad Prism 8.

### Antiviral Assays

The antiviral assay against PRRSV was conducted with rTA-12 M and rTA-Gluc2, and four compounds were tested in this study, including human IFN-α2b, ribavirin, 5-Fluorouracil, and Chloroquine. The 50% inhibition concentration (IC50) was determined to assess their antiviral effect on PRRSV infection. The confluent MARC-145 cells in a 96-well culture plate were pre-incubated with the serially diluted compound for 2 h and then infected with rTA-12 M or rTA-Gluc2 at a dose of 2000 TCID_50_/well. At 2 hpi, virus inoculums were removed, and the compounds at the indicated concentrations were added back to the cells. At 24 hpi, the virus supernatants were harvested for virus titration and gaussia luciferase assay. IC50 was calculated using the absolute IC50 equation with Graphpad prism 8.

### Serum Neutralization Assay

Serum neutralizing antibody (NAb) titers were determined by neutralization assay with rTA-Gluc2. The 15 pig serum samples collected from piglets immunized with a PRRS MLV vaccine were kindly provided by Professor Demin Cai from Yangzhou University. The serum samples inactivated at 56°C for 30 min were serially diluted with MEM and mixed with an equal volume of rTA-Gluc2 (2000 TCID_50_/mL). After being incubated at 37°C for 1 h, the mixtures (100 μl/sample) were added to the confluent MARC-145 cell monolayers in a 96-well culture plate. At 1 hpi, replaced the mixtures with 150 μl infection medium (MEM supplemented with 2% FBS). At 24 hpi, culture supernatants were harvested for gaussia luciferase assay, while cells were fixed with ice-cold methanol for indirect immunofluorescence assay detection of PRRSV N. The NAb titer of a serum sample was expressed as the reciprocal of the highest dilution of a sample causing a 90% reduction in the gaussia activity or fluorescent focus unit. For each sample, the duplicated dilutions were conducted.

## Results

### The Construction and Recovery of the Recombinant Viruses

Initially, we constructed a full-length cDNA clone of the HP-PRRSV TA-12 strain through a two-step homologous recombination *in vitro* (Figure 1A). To differentiate from the parental virus, a genetic marker of *BsrGI* inactivation in pCMV-TA-12 M was introduced through site-directed mutagenesis. Using this cDNA clone, two cDNA clones were constructed to rescue reporter viruses expressing a Gluc. As shown in Figure 1B, the TA-Gluc1 clone contains a Gluc ORF followed by a TRS6 sequence between ORF1b and ORF2a, while the TA-Gluc2 clone contains a TRS sequence followed by a Gluc ORF between ORF7 and 3′UTR.

**Figure 2:**
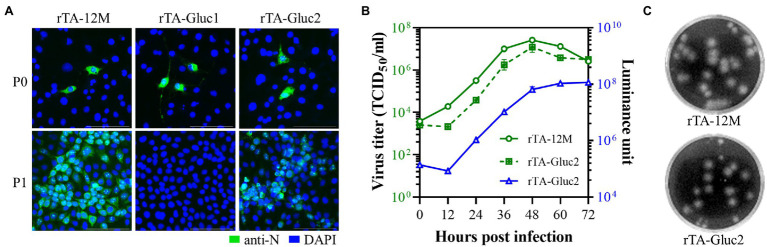
Recovery and *in vitro* characterization of the recombinant viruses. **(A)** Recovery of the recombinant viruses. Briefly, BHK-21 cells in a 12-well plate were transfected with 2 μg cDNA clone plasmid per well using Lipofectamine 3,000 transfection reagent according to the manufacturer’s instructions. At 48 hpt, culture supernatant was harvested as passage zero (P0) virus, while cells were fixed for the detection of N protein *via* IFA. The P0 virus was further passaged to MARC-145 cells. When typical CPE was observed, culture supernatant was harvested as P1 virus, and N protein expression in cells was confirmed by IFA. The nucleus was counterstained with DAPI. Pictures were taken under an epifluorescent microscope. **(B)**The multi-step virus growth curves. MARC-145 cells in a 24-well plate were infected with recombinant viruses of P3 at an MOI of 0.01, respectively. Virus supernatants harvested at 0, 12, 24, 36, 48, 60, and 72 hpi were titrated by TCID_50_ assay (left *Y* axis) and luciferase assay (right *Y* axis). Each data point represents the mean ± deviation of duplicates. **(C)** Plaque morphology of the recombinant viruses. MARC-145 cells were infected with serial diluted recombinant viruses and covered with 1% low melting point agarose supplemented with 2% FBS. At 3 days post-infection, the plaques formed by virus infection were visualized by crystal violet staining.

The recombinant viruses were rescued by DNA transfection of BHK-21 cells. At 48 hpt, N protein expression was detected by IFA in cells transfected with WT and Gluc clones (Figure 2A). The harvested virus supernatants were further passaged to MARC-145 cells, respectively. As expected, the typical cytopathic effect (CPE) was observed in cells infected with the WT virus (rTA-12 M) at 3 dpi. For the Gluc expressing recombinant viruses, CPE was observed in cells infected with rTA-Gluc2 but not in cells infected with rTA-Gluc1. In line with CPE observation, N protein expression was confirmed in cells infected with rTA-12 M and rTA-Gluc2, but not rTA-Gluc1 (Figure 2A). The recombinant viruses were also verified by sequencing the viral genomic regions covering the genetic marker and the reporter gene (data not shown).

### *In vitro* Characterization of the Recombinant Viruses

Next, a multiple-step growth curve was performed to characterize the recombinant viruses. Virus supernatants harvested at 0, 12, 24, 36, 48, 60, and 72 hpi were titrated in MARC-145 cells, while the secreted Gluc in those supernatants was detected by luciferase assay. Based on virus titration results, the WT virus and rTA-Gluc2 exhibited similar growth trends and reached their peak titers (10^7.4^ TCID_50_/mL and 10^7.1^ TCID_50_/mL) at 48 hpi, although virus titers of rTA-Gluc2 were about 0.5 logs lower than those of the WT virus (Figure 2B). Their similar growth abilities were also confirmed by plaque-forming assay (Figure 2C). A gradually increased Gluc activity was detected in virus supernatants of rTA-Gluc2, suggesting that Gluc was expressed during rTA-Gluc2 infection and secreted outside the infected cells. Of note, Gluc production is correlated with virus titers (Figure 2B), indicating that Gluc could serve as an indicator of virus replication.

### The Genetic Stability of rTA-Gluc2 During Serial Passages in MARC-145 Cells

**Figure 3:**
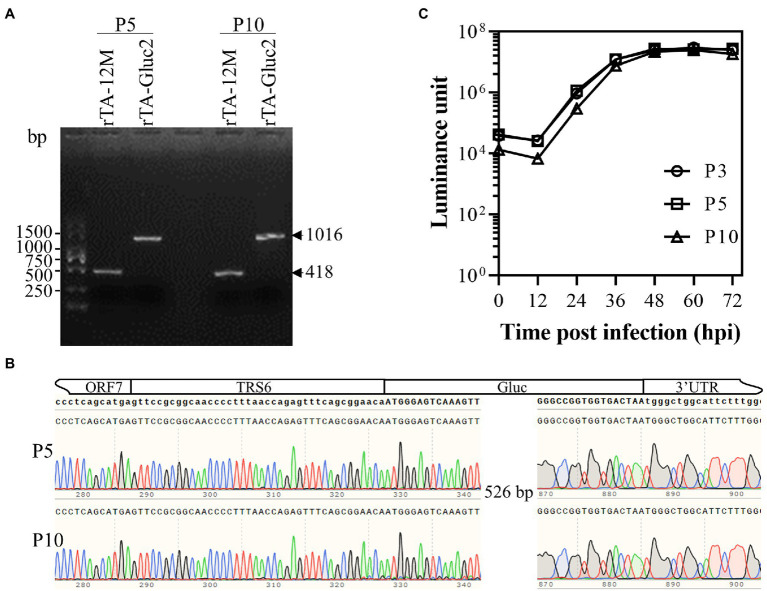
The genetic stability of the Gluc-expressing reporter virus. The Gluc expression cassettes in the recombinant viruses of P5 and P10 were checked by RT-PCR **(A)** and DNA sequencing **(B)**, whereas the corresponding regions in the parental viruses were amplified as a control. In addition, the Gluc expression levels by the recombinant viruses of P3, P5, and P10 were evaluated using the multiple-step growth curves **(C)**. MARC-145 cells were infected with the recombinant viruses at an MOI of 0.01, culture supernatants were harvested at 12, 24, 36, 48, 60, and 72 hpi and titrated by luciferase assay. Each data point represents the mean ± deviation of triplicates.

rTA-Gluc2 has been serially passaged ten times in MARC-145 cells. To determine the genetic stability of the reporter virus, the Gluc expression cassettes of P5 and P10 viruses were amplified by RT-PCR using primer pair, PRRSV-F1/PRRSV-R (Table 1), and the corresponding regions of rTA-12 M P5 and P10 were amplified as controls. As expected, the sizes of the amplified viral genomic regions of rTA-Gluc2 viruses were larger than those of rTA-12 M viruses (Figure 3A). DNA sequencing results with the purified DNA fragments confirmed that no mutation in the Gluc expression cassette was observed (Figure 3B). The growth kinetics of the serially passaged rTA-Gluc2 were further evaluated using the P3, P5, and P10 viruses, and virus replication was indicated by Gluc activity. Consistent with the sequencing results, all three viruses exhibited a very similar growth trend (Figure 3C). Therefore, the reporter virus expressing Gluc remained stable during passages *in vitro*.

### Antiviral Assay Against PRRSV Using rTA-Gluc2 Reporter Virus

**Figure 4:**
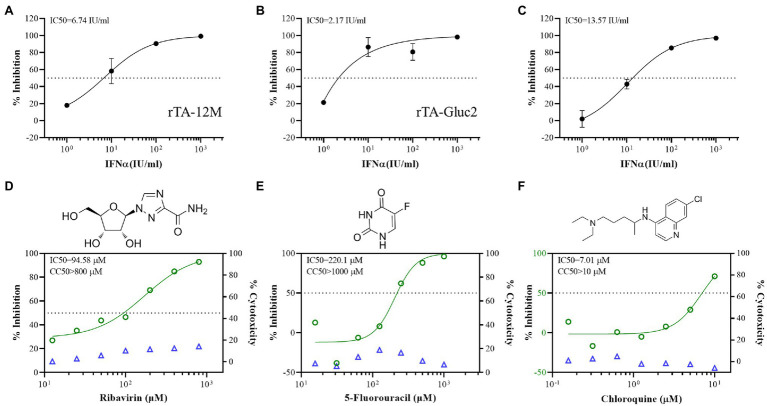
The antiviral assay using the Gluc reporter virus. **(A,B)** The inhibitory effect of IFN-α on the infection of rTA-12 M and rTA-Gluc2 based on virus titer (TCID_50_). **(C)** The inhibitory effect of IFN-α on the infection of rTA-Gluc2 based on gaussia luciferase activity. **(D–F)** The inhibitory effect of the compounds on the infection of rTA-Gluc2 based on gaussia luciferase activity. The inhibition rate is the reduction percentage of viral replication compared to the no treatment control. Each data point represents the mean ± deviation of triplicates.

The reporter virus expressing fluorescent protein is a useful tool for antiviral drug screening. Here, rTA-Gluc2 was explored as a reporter virus for the antiviral screening assay against PRRSV. Since PRRSV is sensitive to type I IFN (Lee et al., 2004), we initially compared the sensitivity of rTA-12 M and rTA-Gluc2 to IFNα. Based on virus titration results, the replication of these two viruses was similarly inhibited by serially diluted IFNα which was indicated by the IC50 doses of 6.74 IU/ml and 2.17 IU/ml against rTA-12 M and rTA-Gluc2, respectively (Figures 4A-B). Besides, based on the inhibition of Gluc activity by IFNα treatment, the IC50 dose against rTA-Gluc2 was 13.57 IU/ml. Thus, rTA-Gluc2 can be used for the antiviral assay, and the Gluc activity is a good indicator of virus replication.

Next, we further evaluated the anti-PRRSV effect of three drugs using rTA-Gluc2, including ribavirin, 5′-Fluorouracil, and chloroquine. The cell cytotoxicity of those three drugs was tested in MARC-145 cells, and no significant cytotoxic effect was observed for all three drugs at the tested doses (Figures 4D-F). For the antiviral assay, the Gluc activity in virus supernatants was detected by luciferase assay, and the IC50 doses were calculated based on the dose–response-inhibition curves. Similar to previous reports (Khatun et al., 2015; Tian and Meng, 2016), ribavirin and 5-Fluorouracil inhibited the infection of reporter virus with the IC50 doses at 94.58 and 220.1 μM (Figures 4D-E), respectively. Chloroquine, an anti-malaria drug, was identified as a broad-spectrum antiviral drug against many RNA viruses, such as SARS-CoV-2 (Liu et al., 2020), human respiratory syncytial virus (Rahman et al., 2021), and hantavirus (Vergote et al., 2021). We also observed the inhibitory effect of chloroquine against PRRSV. Of note, the IC50 of chloroquine is 7.01 μM which is much lower than those of ribavirin and 5-Fluorouracil (Figure 4F), suggesting that chloroquine and its derives are potential drugs against PRRSV. The reduction of virus infection in those antiviral assays was also confirmed by IFA detection of N protein (Figure S1).

### Detection of NAb Against HP-PRRSV Using rTA-Gluc2 Reporter Virus

**Figure 5:**
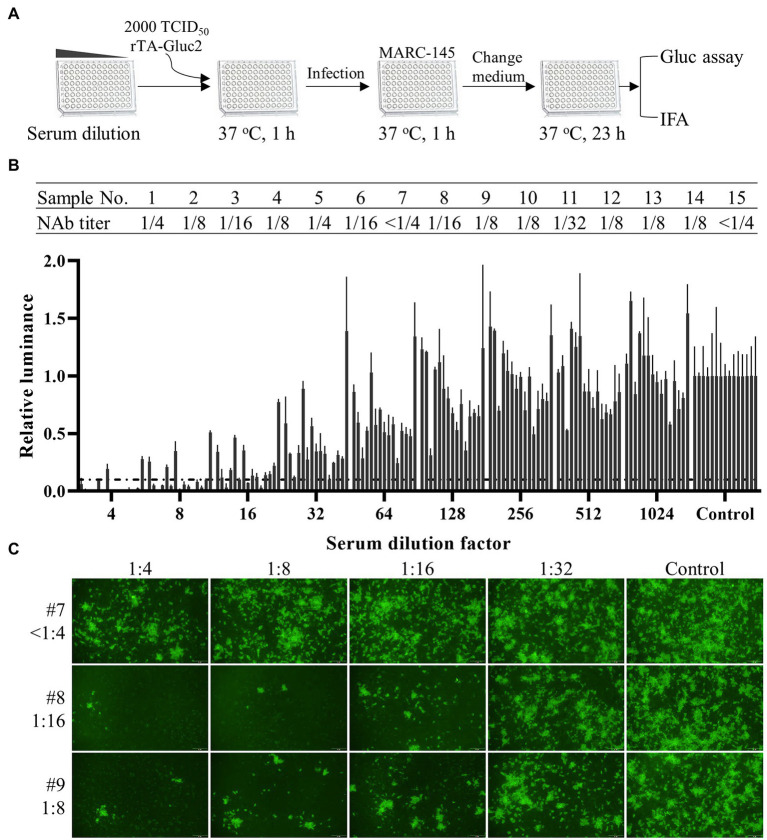
Serum neutralization assay using the Gluc reporter virus. **(A)** A schematic diagram of the procedure for serum neutralization assay. **(B)** The titers of neutralizing antibodies in pig serum samples were calculated based on the reduction of gaussia luciferase activity. Each data point represents the mean ± deviation of duplicates. **(C)** The neutralization of virus infection by selected serum samples was confirmed by IFA detection of N protein expression.

The titer of neutralizing antibodies (NAb) is usually evaluated to monitor the protective immune responses against PRRSV provided by vaccination. For the classical serum neutralization assay (Ostrowski et al., 2002), the NAb titer of a serum sample was expressed as the reciprocal of the highest dilution of a sample causing a 90% reduction in fluorescent focus units (FFU). Although being widely used in diagnostic laboratories, this assay has the following drawbacks: (1) The PRRSV specific antibody used for IFA detection is expensive; (2) It takes several hours to complete the assay, which is time-consuming; (3) An experienced researcher and an epifluorescent microscope are required to accurately record the results. Here, we tried to simplify the NAb detection process using the reporter virus. Since Gluc expressed during viral infection is secreted into viral supernatant, the dynamic of viral replication can be continuously monitored by harvesting a small volume of viral supernatant for luciferase assay. Based on that, a Gluc readout-based NAb detection procedure was designed as shown in Figure 5A. According to this procedure, the NAb titers of 15 pig serum samples were evaluated. As shown in Figure 5B, the NAb titers of 13 samples were between 1/4 and 1/32, whereas no NAb (<1/4) was detected in two samples. We also confirmed the NAb titers of all samples by IFA detection of N protein. In Figure 5C, the NAb titers of three selected samples (#7, #8, and #9) determined by FFU reduction were consistent with those determined by luciferase activity reduction.

## Discussion

A reverse genetics system is a valuable tool for the investigation of RNA viruses in many aspects. Taking advantage of this system, the research on PRRSV molecular biology has made significant progress in understanding the mechanisms of viral RNA synthesis, the function study of viral proteins, and the identification of viral virulence factors and viral determinants of cell tropism (reviewed in (Chaudhari and Vu (2020)). With the reverse genetics of PRRSV available, PRRSV was also explored as a viral vector for the expression of a foreign gene of interest (Fang et al., 2006; Pei et al., 2009; Zheng et al., 2010; Guo et al., 2016; Gao et al., 2018; Huang et al., 2018; Wang et al., 2020). In this study, an infectious cDNA clone of the HP-PRRSV TA-12 strain was established *via* homologous recombination *in vitro*. Through reverse genetics manipulation, we generated a reporter virus stably expressing Gluc in which the Gluc gene was inserted between ORF7 and 3′UTR. *In vitro* characterization showed that Gluc expression level in culture media of reporter virus-infected cells is a good indicator of virus progeny. Based on that, this reporter virus was also applied for the antiviral drug screening assay and SNA using Gluc activity as readout.

Several PRRSV reporter viruses expressing fluorescent proteins and luciferases have been generated for tracking viral infection *in vitro* (Pei et al., 2009; Sang et al., 2012; Wang et al., 2020). As the most variable region in the PRRSV genome, nsp2 serves as a good target for foreign gene insertion. For instance, a study using a recombinant PRRSV expressing EGFP-tagged nsp2 to track intercellular traffic of nsp2 revealed a novel nanotube-based transmission mechanism between cells (Guo et al., 2016). However, the genetic instability of the reporter genes in the PRRSV genome is an issue to be addressed. Besides insertion in nsp2, the other three potential locations have been explored to express a reporter gene as a dedicated expression cassette, including between ORF1b and ORF2a (Pei et al., 2009), between ORF4 and ORF5a (Wang et al., 2020), and between ORF7 and 3′UTR (Wang et al., 2013). The reporter genes inserted between ORF1b and ORF2a and between ORF7 and 3′UTR usually remained stable for several passages, although the genetic stability of the reporter genes seems to depend on the nature of the reporter genes. In this study, we tried to generate recombinant viruses expressing Gluc through a dedicated expression cassette which was inserted between ORF1b and ORF2a or between ORF7 and 3′UTR as described previously (Pei et al., 2009; Wang et al., 2013). The reporter virus with Gluc insertion between ORF7 and 3′UTR was successfully recovered and exhibited similar growth kinetics to the WT virus (Figure 2). This reporter virus remained genetically stable for at least ten passages *in vitro* (Figure 3). However, we failed to rescue the reporter virus that contains a Gluc expression cassette between ORF1b and ORF2a, although N protein expression was detected in BHK-21 cells transfected with the cDNA clone (Figure 2A). In line with our results, Zheng et al. (2010) reported that the PCV2 cap gene was deleted from the PRRSV genome at passage 1 when they tried to rescue a recombinant virus expressing this gene *via* a dedicated expression cassette between ORF1b and ORF2a. Thus, our results further support the scenario that the nature of the reporter gene and insertion location within the PRRSV genome are important for the viability and genetic stability of the reporter virus.

The classical method for antiviral drug screening and serum neutralizing antibody detection are plaque reduction-based or fluorescent focus unit reduction-based tests, which are time-consuming, labor-intensive, and expensive. Alternatively, PRRSV reporter viruses have been utilized in the antiviral screening assay using the fluorescent signal as a readout (Huang et al., 2018). However, the autofluorescence of samples, vessels, and imaging media may reduce the assay accuracy, and an experienced researcher is also required to record results. To generate a recombinant PRRSV reporter virus for antiviral drug screening and SNA, we chose Gluc as the reporter gene because of its small size, unique thermal stability, and genetically encoded secretion system. To ensure that the Gluc levels in culture media released by the reporter virus can indicate viral infection, we determined the dynamic of Gluc secretion in culture media. As expected, the secreted Gluc levels are correlated with virus progenies (Figure 2B). Based on these results, the rTA-Gluc2 was utilized for the antiviral screening assay. Using this reporter virus, we confirmed the anti-PRRSV effect of IFNα, ribavirin, and 5-Fluorouracil (Figures 4A-E). Furthermore, chloroquine which is a compound with a broad antiviral spectrum also exhibited an inhibitory effect on PRRSV infection at a very low concentration. The classical SNA for PRRSV neutralizing antibody detection used in diagnostic laboratories is a fluorescent focus unit reduction-based method (Ostrowski et al., 2002). In this assay, IFA with PRRSV-specific antibody is conducted to detect virus infection, which is time-consuming. An experienced researcher is required to record the neutralizing antibody titers. We established a Gluc readout-based SNA using rTA-Gluc2, which generated similar results as the classical SNA (Figure 5). Due to the secretion nature of Gluc, the Gluc activity in culture media was evaluated to access the reduction of virus infection without cell lysis. This Gluc readout-based SNA does not require experienced researchers to record results and could save several hours for IFA detection. This novel SNA is also applicable for the high-throughput format. Taken together, the recombinant PRRSV expressing Gluc serves as a nice tool for antiviral drug screening and neutralizing antibody detection.

In conclusion, we established reverse genetics for HP-PRRSV TA-12 strain *via* homologous recombination *in vitro* and generated a recombinant PRRSV stably expressing Gluc. Using this reporter virus, the Gluc readout-based assays for antiviral drug screening and neutralizing antibody detection were established.

## Data Availability Statement

The original contributions presented in the study are included in the article/supplementary materials, further inquiries can be directed to the corresponding author.

## Author Contributions

YL: conceptualization, supervision, founding acquisition, and wrote the original draft. YL and CR: investigation. CR, CL, and YZ: methodology, formal analysis, and data curation. YL, CL, and YX reviewed and edited the manuscript. YX: contributed to the resources. All authors contributed to the article and approved the submitted version.

## Funding

This project was supported by the National Natural Science Foundation of China (grant nos. 31902254 and 32072833), Jiangsu Agricultural Science and Technology Innovation Fund (grant no. CX(21)3125), Jiangsu Co-innovation Center for Prevention and Control of Important Animal Infectious Diseases and Zoonoses, and the Priority Academic Program Development of Jiangsu Higher Education Institutions (PAPD). YL is supported by the Scientific Research Foundation of Yangzhou University and the “LvYangJinfeng Program” of Yangzhou City.

## Conflict of Interest

The authors declare that the research was conducted in the absence of any commercial or financial relationships that could be construed as a potential conflict of interest.

## Publisher’s Note

All claims expressed in this article are solely those of the authors and do not necessarily represent those of their affiliated organizations, or those of the publisher, the editors and the reviewers. Any product that may be evaluated in this article, or claim that may be made by its manufacturer, is not guaranteed or endorsed by the publisher.

## Footnotes

^1^ https://talk.ictvonline.org/taxonomy/
