# Supplementary material for: A Recombinant Porcine Reproductive and Respiratory Syndrome Virus Stably Expressing a Gaussia Luciferase for Antiviral Drug Screening Assay and Luciferase-Based Neutralization Assay
Source: Front Microbiol. 2022 May 13;13:907281. doi: 10.3389/fmicb.2022.907281 (PMC9136234; doi:10.3389/fmicb.2022.907281)
Supplement: Supplementary file 1 [file Image_1.pdf]

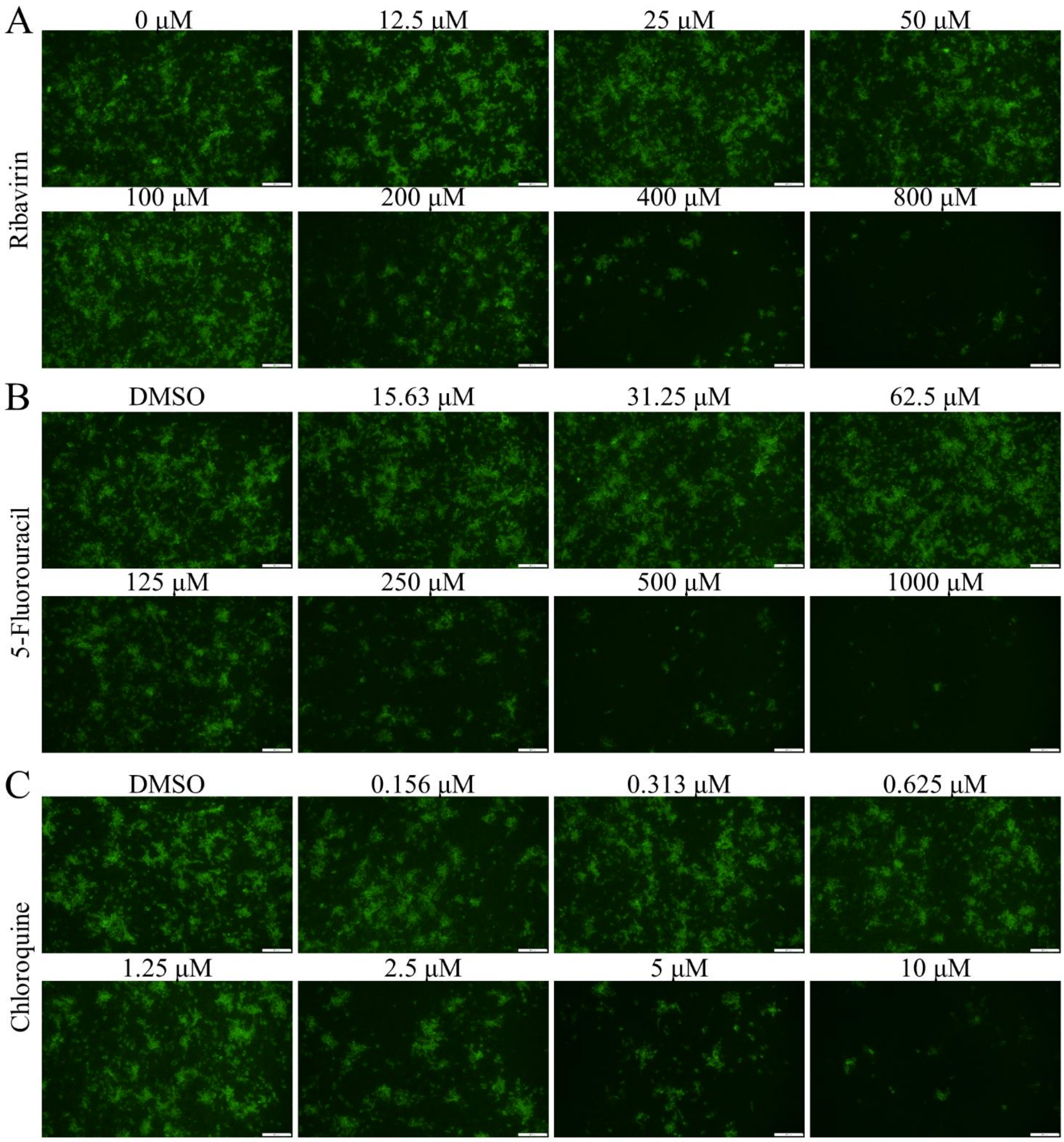

Figure S1. The inhibitory effects of three tested drugs on rTA-Gluc2 infection determined by IFA detection of N protein expression.
